# Supplementary material for: Evaluating the Sensitivity of Heat Wave Definitions among North Carolina Physiographic Regions
Source: Int J Environ Res Public Health. 2022 Aug 16;19(16):10108. doi: 10.3390/ijerph191610108 (PMC9408726; doi:10.3390/ijerph191610108)
Supplement: Supplementary file 1 [file ijerph-19-10108-s001.zip › ijerph-1842872-SI.pdf]

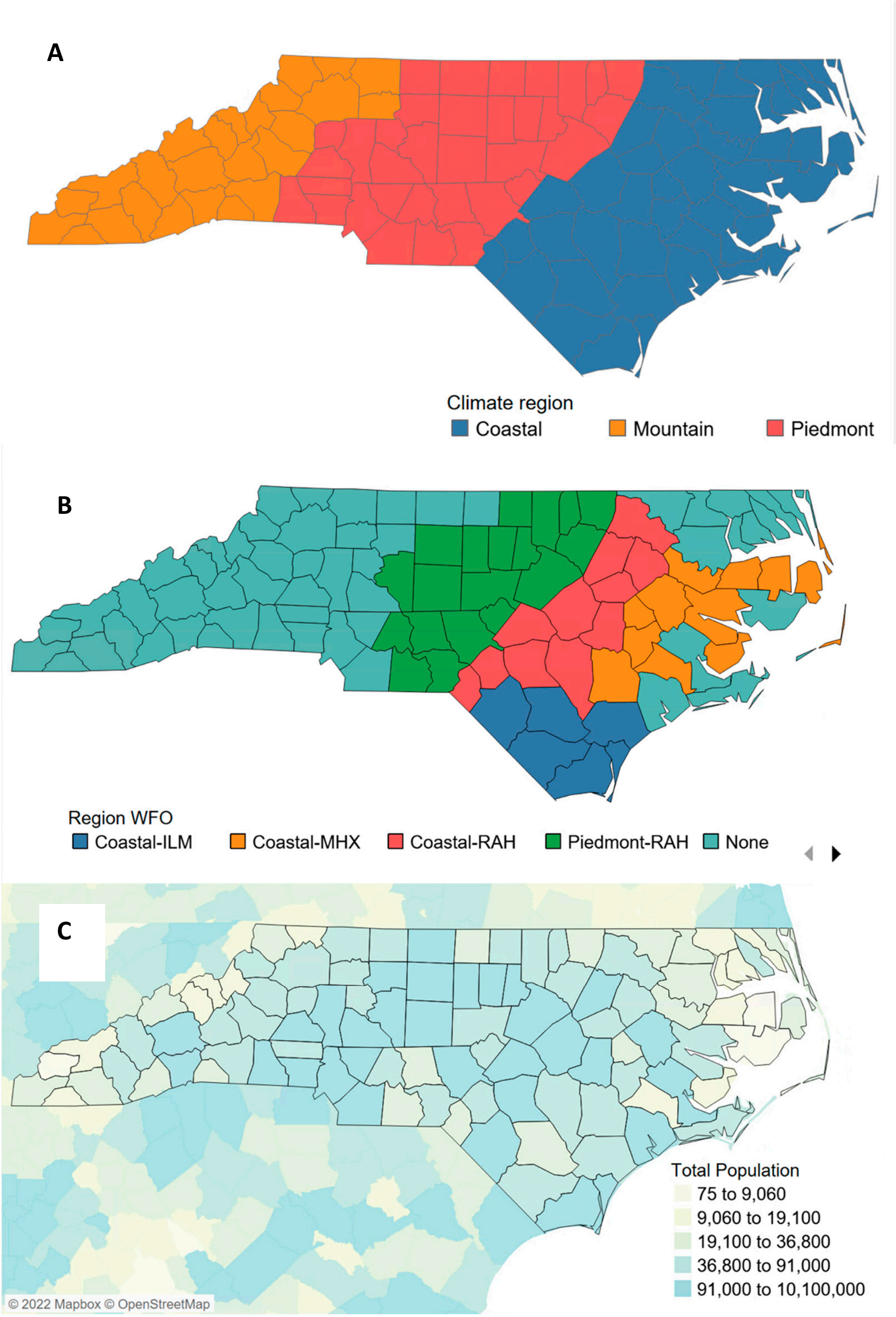

Figure S1. North Carolina physiographic regions. A. Counties clustered by physiographic region. B-WFOs within physiographic regions included in this study. C-Total population per North Carolina Counties.
